# Influence of radiotherapy on cardiac implantable devices and leads—a single-institution analysis and critical evaluation of current guidelines

**DOI:** 10.1007/s00066-024-02345-0

**Published:** 2025-01-10

**Authors:** Jakob Warmbrunn, Christoph Straube, Hans-Ulrich Haase, Daniel Sinnecker, Karl-Ludwig Laugwitz, Stephanie E. Combs, Simon Schneider, Daniel Habermehl

**Affiliations:** 1 TUM School of Medicine and Health, Department of Radiation Oncology, Technische Universität München (TUM), Klinikum rechts der Isar, Munich, Germany; 2 TUM School of Medicine and Health, Department of Internal Medicine I, Technische Universität München (TUM), Klinikum rechts der Isar, Munich, Germany; 3 MVZ Harz, Goslar, Germany; 4 Department of Radiation Sciences (DRS), Helmholtz Zentrum München, Institute of Innovative Radiotherapy (iRT), Munich, Germany; 5 German Consortium for Translational Cancer Research (dktk), Partner Site Munich, Berlin, Germany; 6 Department of Radiation Oncology, Giessen-Marburg University Hospital, Giessen, Germany

**Keywords:** Cardiac implantable electronic devices, Radiation therapy, Pacemaker dependency, Device malfunctions, Neutron-producing radiation

## Abstract

**Purpose:**

Increasing life expectancy and advances in cancer treatment will lead to more patients needing both radiation therapy (RT) and cardiac implantable electronic devices (CIEDs). CIEDs, including pacemakers and defibrillators, are essential for managing cardiac arrhythmias and heart failure. Telemetric monitoring of CIEDs checks battery status, lead function, settings, and diagnostic data, thereby identifying software deviations or damage. This study evaluates the German Society for Radiation Oncology (DEGRO)/German Society for Cardiology (DGK) guideline, assessing real-world complications and risk factors and analyzing pacemaker and implantable cardioverter-defibrillator (ICD) lead function for their predictive value concerning device malfunction.

**Methods:**

A total of 54 patients with pacemakers or ICDs who underwent radiation therapy were identified. Demographics, treatment courses, and device information from physical and digital records were extracted. DEGRO/DGK risk groups and pacemaker dependency at the start of RT were assessed. Delineation of the devices and lead insertion sites was performed in the treatment planning system. Dosimetric information from the treatment plans was then correlated with reports of standardized device checks.

**Results:**

Over 80% of patients were treated with dual-chamber pacemakers or cardiac resynchronization therapy (CRT), and 16.7% had ICDs. One third of patients were pacemaker dependent. 59.3% of patients were in the low-risk category, 29.3% in the medium-risk, and 11.1% in the high-risk category. Thoracic irradiation resulted in the highest median dose to devices. Lead parameter deviations exceeding thresholds were found in 14.8% for the stimulation threshold and 13.5% for sensing. Device malfunctions occurred in 3.7% of cases, both involving electrical resets and neutron-producing radiation (beam energy 10 megaelectron volt (MV) or higher).

**Conclusion:**

Collecting lead parameters in addition to secure malfunctions like electrical restarts and memory erasure revealed a significant proportion of treatment courses with temporary changes, though no correlation with individual treatment plans or adverse events was found. The focus on reducing neutron-producing radiation could be further supported.

## Introduction

With an increasing life expectancy and advances in cancer diagnosis and treatment, a rise in the number of patients requiring both radiation therapy and a cardiac implantable electronic device (CIED) is anticipated [1]. CIEDs, encompassing pacemakers (PMs), cardiac resynchronization therapy (CRT), and implantable cardioverter-defibrillators (ICDs), are crucial for managing cardiac arrhythmias and heart failure. Telemetric monitoring of CIEDs allows regular assessment of parameters such as battery status, lead function, programmed settings, and diagnostic data [2]. Deviations in software integrity can be identified during telemetric checks as an error message or the presence of a “backup” mode. Physical damage to pacemakers and ICD leads from MRI imaging has been extensively studied. Heating of the lead material appears to play the main role in lead damage [3]. Corresponding studies on the impact of radiation therapy on leads and the tissue into which they are inserted are lacking. Cardiac irradiation frequently results in long-term myocardial fibrosis. Additionally, a local dose increase in the vicinity of metallic implants has been described [4, 5]. These structural changes in the myocardium lead to decreased tissue resistance, which could manifest as a reduction in lead impedance and an increase in pacing threshold [6].

This study evaluates the current guideline of the German Society for Radiation Oncology (DEGRO) and the German Society for Cardiology (DGK) [7] regarding practicality in clinical settings. We assess real-world complications rates and explore risk factors beyond device type and radiation dose. Furthermore, detailed data on pacemaker and ICD leads and their insertion sites were collected and analyzed for their predictive value or possible influence on device malfunction.

## Methods

### Patient characteristics

Fifty-four patients with pacemakers or ICDs who underwent radiation therapy between 2001 and 2016 at the Klinikum rechts der Isar, Munich, were identified from a database. From the physically and digitally archived records, patient characteristics and treatment courses were extracted. The standardized reports of device checks provided information about the implanted devices in addition to therapy-specific data. The DEGRO/DGK risk group was determined at the start of therapy. Pacemaker dependency was defined by an intrinsic heart rate of less than 30 beats per minute. Patient demographics, treatment courses, and device information were extracted from physical and digital medical records. Standardized device check reports provided therapy-specific data.

### Structures of interest and dosimetry

The implanted devices and the lead insertion sites were delineated separately in the treatment planning system. Quality control was performed by comparing the contoured volumes of the devices with the manufacturer’s specified volumes and through evaluation by experienced radiation therapists.

The delineated structures of interest in this study were

- device bodies: complete, pectorally implanted device;
- lead insertion sites: lead tip with thread, anchored in the working myocardium;
- localization: atrium, right ventricle, left ventricle.

A 0.5-cm margin was generated around the structure, and dosimetric calculations were performed. This accounted for thoracic respiratory excursions and potential positional uncertainties. Dosimetric data were extracted from the treatment planning system (Eclipse v 13.5 and 15.6, Varian Medical Systems, Palo Alto, California, USA and Accuray Precision v1.1 and 2.0, Accuray Inc., Sunnyvale, Califonia, USA). The following parameters were extracted from the plans:

- minimum dose (Dmin): the minimum radiation dose in Gy that the structure receives within the volume;
- maximum dose (Dmax): the maximum radiation dose in Gy that the structure receives within the volume;
- mean dose (Dmean): the average radiation dose related to the volume of the structure;
- median dose (Dmedian): the median radiation dose related to the entire volume of the structure.

For the evaluation of device data, a timepoint up to 2 weeks before or immediately at the beginning (after the first fraction) of radiation therapy and a timepoint at the end (last radiation week) or shortly after radiation therapy were chosen. Threshold analysis based on established literature (comparable to MRI studies on CIEDs) was performed to assess clinically relevant changes.

### Statistical analysis

Ethical approval was waived by the local Ethics Committee of the Technical University Munich (TUM) in view of the retrospective nature of the study and the fact that all procedures performed were part of the routine care.

The data were collected using Microsoft Excel Version 16.0 (Microsoft Corp., Redmond, Washington, USA) and statistically and graphically analyzed with IBM SPSS Statistics 23 (International Business Machines Corporation, Armonk, New York, USA). The present cases were primarily analyzed descriptively. The limited number of cases, as well as the low event rate, did not allow for a more in-depth statistical evaluation. Using non-parametric tests (Spearman’s correlation, Mann–Whitney U test, and Kruskal–Wallis H test), no significant correlation could be established between the adverse event rate and cumulative dose, irradiation energy, or region of irradiation. A chi-square test was used to examine whether adverse events occur more frequently in relation to the exceedance of lead parameter thresholds.

## Results

### Indications for RT and CIED

Information on patient characteristics is provided in Table 1. Over 80% of all patients were treated with a dual-chamber pacemaker or CRT. Nine patients (16.7%) had an ICD, and one patient had a combination of ICD and CRT (CRT-D). One-third of the patients were pacemaker dependent, with an intrinsic heart rate of less than 30 beats per minute. The average age of the CIEDs at the start of therapy was 3 years (36 months; range 2 weeks–8 years).

**Table 1:** Patient characteristics

Characteristic	*N*	Percentage (%)
Age (years)^a^	74	51–89
Age of implanted device (months)^a^	36	0.5–101
*Gender*		
Male	39	72.2
Female	15	27.8
*Implanted device*		
Pacemaker	43	79.6
ICD	9	16.7
CRT	1	1.9
CRT‑D	1	1.9
*Device dependency*		
Non-dependent	36	66.7
Dependent	18	33.3
*Manufacturer*		
Medtronic	28	51.8
Biotronik	16	29.6
St. Jude Medical	7	13.0
Cardiac pacemakers, Inc. (CPI)	3	5.6
*Irradiated region*		
Head	12	22.2
Neck	7	13.0
Thorax	17	31.5
Esophagogastric junction	3	5.6
Abdomen	2	3.7
Pelvis	13	3.7

^a^Data provided as mean and range Abbreviations: *ICD* Implantable Cardioverter Defibrillator, *CRT* Cardiac Resynchronization Therapy, *CRT-D* Cardiac Resynchronization Therapy Defibrillator; Manufacturers: Medtronic (Dublin, Ireland), Biotronik (Berlin, Germany), St. Jude Medical (St. Paul, MN, USA), Cardiac Pacemakers, Inc. (St. Paul, MN, USA)

The majority of CIEDs were from Medtronic (Medtronic plc, Dublin, Ireland; 51.8%), Biotronik (BIOTRONIK SE & Co. KG, Berlin, Germany; 29.6%), and St. Jude Medical (St. Jude Medical, Inc., Little Canada, Minnesota, USA; 13%). Only 5.6% of CIEDs were from CPI (CPI International, Inc., Palo Alto, California, USA).

### Dosimetric analysis

In the cohort of patients analyzed, 35 (65%) were treated with a radiation energy of 6‑MV photons, 7 (13%) with 10-MV photons, and 12 (22%) with 15-MV photons. According to the subsequently published DEGRO/DGK guideline, radiation energy should be limited to a maximum of 6‑(10-)MV photons. Although exceeding this limit does not preclude risk classification in the guideline, it should be noted that a higher radiation energy was used in our cohort. Therefore, a generally higher risk of malfunction must be assumed than suggested by the risk group classification. None of the patients with an ICD had a history of prior ventricular fibrillation, so this characteristic had no influence on the risk group classification.

A total of 59.3% of the group were retrospectively assigned to a low-risk category for an event (no pacemaker dependency, dose < 2 Gy). A total of 29.3% of the patients were in the medium-risk group (pacemaker dependency and < 2 Gy or 2–10 Gy). This was mainly due to pacemaker dependency (81.2%) and less due to the dose to the device (18.8%).

In total, 6 of the 54 patients (11.1%) were assigned to the high-risk group (pacemaker dependency and > 2 Gy or > 10 Gy).

Most patients (31%) in the study group received radiation to the thorax, followed by the pelvis (24%). Overall, a total of 22% of patients received RT to the head region and 13% to the neck. The gastroesophageal junction was treated as a separate region and was irradiated in three patients (6%). Only two patients (4%) were irradiated in the abdominal region.

The mean cumulative dose to the planning target volume (PTV) was 53 Gy (21 Gy to 76.5 Gy). The dose to the device was retrospectively determinable in 24 (44.4%) patients. In 8 (14.8%) patients, retrospective dose determination was not feasible due to technical issues. For 22 (40.74%) patients, the implanted device was located outside the irradiated area as captured by the planning CT. Significant differences in the cumulative dose to the device emerged depending on the location of the device relative to the PTV and the technical possibility of sparing the device.

The mean Dmean at the pacemaker was 0.95 Gy (median 0.32 Gy), ranging from barely measurable (0.003 Gy) to 5.35 Gy. The mean Dmax was 2.86 Gy (median 1.3 Gy; maximum 14.88 Gy). Mean Dmean per fraction was 34 mGy (0.15 to 192 mGy), mean Dmax per fraction was 104 mGy (1 to 604 mGy).

As expected, thoracic irradiation resulted in the highest median dose (1.82 Gy; range 0.18–14.88 Gy) to the devices. Maximum cumulative doses progressively decreased for neck (3.32 Gy), head (1.39 Gy), and gastroesophageal junction (0.84 Gy) irradiation. Pelvic and extremity radiotherapy excluded devices due to spatial distance.

Maximum cumulative doses were also evaluated for lead insertion sites with a 5-mm safety margin. Data were available for 12 patients each in the right atrium and ventricle, and 3 in the left ventricle (or sinus venosus). The mean maximum dose was highest in the left ventricle (19.1 Gy; range 0.12–50.66 Gy), followed by the right atrium (13.25 Gy; range 0.046 Gy) and right ventricle (11.95 Gy; range 0.004–42.66 Gy).

### Lead parameters

**Fig. 1:**
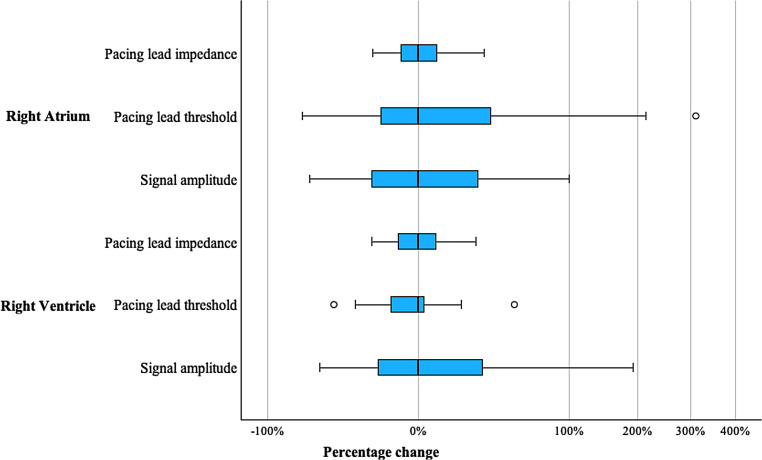
Percentage change in aggregate control parameters during the course of therapy (boxplot)

As shown in Fig. 1, the comparison of parameters over the study period does not indicate a significant change. The greatest percentage variance occurred in the sensing of the right atrium and the right ventricular lead. Non-parametric sign tests revealed no significant short-term trends in control parameters. Table 2 shows the results of the threshold analysis of lead parameters and the analysis of an association between threshold exceedance and adverse effects/high radiation energy using the chi-square test. Lead impedance in both the right and left ventricles remained within expected thresholds. Deviations exceeding thresholds were observed in 14.8% and 13.5% of cases for stimulation threshold and sensing, respectively, with a higher frequency in atrial leads compared to ventricular leads.

**Table 2:** Short term effect of radiotherapy on CIED lead parameters

	*N*	Mean change ± SD	Mean change (%) ± SD	Threshold (%)	Number of leads exceeding threshold, *n* (%)	Differences in threshold violation regarding device malfunction (*p*-value, x^2^ test)	Differences in threshold violation regarding high-LET (*p*-value, x^2^ test)
*Pacing lead impedance*	*–*	*Ohm*	–	–	–	–	–
All leads	50	3.1 ± 40.4	0.9 ± 7.9	30	0 (0)	–	–
Right atrium	21	8.7 ± 45.9	1.9 ± 9.1	30	0 (0)	–	–
Right ventricle	29	0.9 ± 36.2	0.2 ± 7.1	30	0 (0)	–	–
*Pacing lead threshold*	*–*	*V*	–	–	–	–	–
All leads	54	0.1 ± 0.4	9.5 ± 42.6	50	8 (14.8)	–	–
Right atrium	22	0.0 ± 0.3	7.5 ± 38.7	50	5 (22.7)	0.724	0.402
Right ventricle	32	0.1 ± 0.5	10.9 ± 45.7	50	3 (9.7)	0.632	0.296
*Signal amplitude*	*–*	*mV*	–	–	–	–	–
All leads	52	−0.1 ± 1.7	13.9 ± 58.9	40	7 (13.5)	–	–
Right atrium	25	0.2 ± 1.1	30.2 ± 81.4	40	6 (24.0)	0.533	0.734
Right ventricle	27	−0.3 ± 2.1	−1.1 ± 13.7	40	1 (3.7)	0.842	0.398

*high-LET* high linear energy (photon energies > 10 MV) transfer (LET) ionizing radiation (neutron producing)

### Malfunction

Out of 54 treatment series, device malfunction in the form of an electrical reset occurred in 2 (3.7%) cases. In one case, this was indicated by an electrical alarm. In the other case, the reset was retrospectively observed during pacemaker monitoring. Both cases were assigned to the low- and medium-risk groups according to the DEGRO/DGK risk group classification, respectively. Noteworthily, neutron-producing radiation with 10 and 15 MV, respectively, was used in both cases. According to the guideline, which was not available at that time, the radiation energy in the radiation treatment planning should have been limited to 6 (10) MV.

The two cases with events (electrical reset) were examined in detail and the results are presented in Table 3. In the first case, an older pacemaker malfunctioned at a cumulative dose of approximately 0.3 Gy. The relatively young and more complex device of the second patient (ICD combined with CRT) received a cumulative dose of about 1.9 Gy. The high cumulative dose at the lead insertion sites of over 40 Gy is notable. In neither case were there significant changes in the device parameters over time.

**Table 3:** Case description of malfunction (electric reset)

	Case 1^a^	Case 2^a^
*Age, gender*	77, male	83, male
*Risk group (retrospectively)*	Intermediate	Low
*Radiated region*	Esophagogastric junction	Esophagogastric junction
*Cumulative dose PTV*	54 Gy	55.8 Gy
*Beam energy*	15 MV^**b**^	10 MV^**b**^
*Radiation technique*	3D conformal	3D conformal
*Fractions (n)*	29	31
*Cumulative dose CIED (Dmax)*	0.312 Gy	1.899 Gy
*Cumulative dose CIED (Dmax) at the time of the event*	Not available	Approximately 0.5 Gy
*Cumulative dose at lead insertion site*		
RA	7.677 Gy	42.553 Gy
RV	9.752 Gy	35.019 Gy
(LV)	–	(27.938 Gy)
*CIED*	Pacemaker	ICD + CRT
*Device dependent*	Yes	No
*History of prior fibrillation*	–	No
*Manufacturer*	Vitatron	Medtronic
*Indication*	AV block	Primary prevention and resynchronization
*Age of device*	2172 days	236 days
*Number of CIED controls*	2	13
*Device parameter changes*	No tendency	No tendency

^a^Prior to publication of the DEGRO/DGK guideline

^**b**^According to the guideline, beam energy should have been limited to 6‑(10-)MV photons Abbreviations: *AV block *Atrioventricular block, *CIED* Cardiac Implantable Electronic Device, *CRT* Cardiac Resynchronization Therapy, *DEGRO* Deutsche Gesellschaft für Radioonkologie, *DGK* Deutsche Gesellschaft für Kardiologie, *ICD* Implantable Cardioverter Defibrillator, *LV* Left ventricle, *MV* Megaelectronvolt, *PTV* Planning Target Volume, *RA* Right atrium, *RV* Right ventricle; Manufacturers: Medtronic (Dublin, Ireland), Vitatron (Maastricht, Netherlands; later acquired by Medtronic)

## Discussion

Balancing the risk of cardiac implantable electronic device (CIED) malfunction with oncological needs is crucial during RT. Guidelines like the DEGRO/DGK aim to optimize this balance [7]. Often, guidelines for rare diseases and clinical issues are based more on expert opinions than on controlled studies [8]. This explains differences between guidelines, for example between the DEGRO/DKS and the 2022 ESC guidelines [17]. For example, the surveillance recommendations of the ESC guideline in the high-risk group are weekly device checks, electrocardiography (ECG) or pulse oximetry monitoring during RT, and the presence of an external pacemaker [9]. In contrast, the DEGRO/DGK guideline recommends daily readouts in case of high-risk indications. Given the low number of events as well the absence of fatal events in almost all reported series, longer surveillance intervals, as recommended by the ESC guidelines, might be acceptable [10]. It is important to note that in the years following the publication of the German guidelines, substantial evidence was generated regarding the occurrence of malfunctions, which justifies a less stringent monitoring of patients.

### Cumulative dose to the device as a risk factor for adverse events

A dose–effect relationship is documented in the literature. Mouton et al. (2002) reported a cumulative dose of less than 2 Gy leading to 11.5% of the devices experiencing “relevant malfunction.” The study concludes that there is no safe dose threshold for irradiating an implanted device [11]. Potential malfunctions described by Hurkmans et al. (2005) include unintended changes in pulse amplitude, frequency, and threshold; loss of telemetry capability; complete functional failure; and other issues (battery depletion, lead impedance change). These malfunctions occurred with direct irradiation of pacemakers at 5 Gy or higher [12]. Furthermore, ICDs appear to be more sensitive to low cumulative doses due to more complex circuits [13]. Controversially, in vivo studies often yield different results: Grant et al. (2015) conducted a retrospective analysis of 249 irradiations in patients with CIEDs. No correlation between the cumulative dose and malfunctions was found for a device up to 30 Gy [14].

In the present study, the maximum cumulative dose to the device for both malfunctions was below 1 Gy. Devices with significantly higher doses showed no malfunction. Although the cumulative dose to the device is the most extensively studied risk factor for malfunction, the threshold of 2 Gy in the current DEGRO/DGK risk stratification seems relatively restrictive. In the Heart Rhythm Society (HRS) Expert Consensus Statement of 2017, involving American, European, Asian, and Latin American societies, it is recommended to consider relocating the device above a cumulative dose of 5 Gy. Regular CIED checks are recommended, especially in cases of neutron-producing irradiation and pacemaker dependency of the patient [15].

### Radiation energy as the main factor in risk stratification

The DEGRO/DGK guideline mentions the energy level of the radiation as one of the key risk factors and recommends limiting it to 6 (10) MV. It is not mentioned that classification into risk groups outside of these limitations is not recommended; however, it should be noted that limiting the radiation energy to 6 MV might have potentially prevented both malfunctions [7]. In newer guidelines, including those of French, Italian, and European societies, neutron-producing irradiation with energies ≥ 10 MV is considered an independent risk factor (Table 4; [9, 16, 17]). In a study from 2020, Gauter-Fleckenstein et al. prospectively evaluated the implementation of the DEGRO/DGK guidelines and compared them with the 1994 APPM guidelines. In this context, the radiation energy used for CIED patients was limited to 6 MV. There were no events in 160 cases. The authors found no significant influence of cumulative dose, irradiated region, PTV or fraction dose, or CIED manufacturer. A tendency towards higher susceptibility to errors in ICDs compared to pacemakers was observed [18].

**Table 4:** Comparison of the guidelines of international societies regarding the management of CIED patients undergoing radiotherapy

Society		DEGRO/DGK Germany, 2015 [4]	AIAC/AIRO/AIFM Italy, 2017 [13]	SFRO France, 2021 [6]	SC (EHA/ESTRO/IC-HS) Europe, 2022 [14]
*Cumulative dose thresholds*		2 Gy	2 Gy	5 Gy	5 Gy
		10 Gy	10 Gy		10 Gy
*Radiation energy thresholds*		6-(10-)MV photons	6 MV	10-MV photons	10-MV photons
*Factors for risk group classification*	Pacemaker dependency	Yes	Yes	Yes	Yes
	ICD: regular shock delivery	Yes	Yes	Yes	Yes
	Difference between pacemaker/ICD	No	Yes	No	No
*Recommendations*^a^	Low risk	Standard measures^a^	Audiovisual monitoring CIED checks before/after the first radiation/halfway through the radiation cycle/end of the radiation cycle 1 month and 6 months after the radiation cycle	CIED check once before/after RT cycle	Audiovisual monitoring CIED check once before/after RT cycle
	Medium risk	CIED checks before/after each radiation ECG/SpO_2_ monitoring	CIED check as for low risk ECG/SpO_2_ monitoring	CIED check weekly	No medium-risk group mentioned
	High risk	Relocation or cardiologist/anesthetist present CIED checks before/after each radiation ECG/SpO_2_ monitoring	Consider relocation Electrophysiologist on standby CIED checks as for low risk, additionally weekly during RT cycle	Consider relocation or ECG/SpO_2_ monitoring Cardiologist/intensivist present Magnet placement if necessary	Relocation or ECG/SpO_2_ monitoring CIED checks before and after RT cycle, and weekly

^a^Standard measures in every risk group: evaluation and CIED check before the first radiation, trained personnel, emergency plan, defibrillator

*AIAC* Associazione Italiana Aritmologica e Cardiostimolazione *AIRO* Associazione Italiana Radioterapia Oncologica, *AIFM* Associazione Italiana Fisica Medica*, SFRO* Société Française de Radiothérapie Oncologique, *ESC* European Society of Cardiology, *EHA* European Heart Association, *ESTRO* European Society for Radiotherapy and Oncology, *IC-HS* International Cardio-Oncology Society, *CIED* Cardiac Implantable Electronic Device, *ECG *Electrocardiogram, *SpO₂* Peripheral Oxygen Saturation, *MV* Megaelectronvolt, *RT* Radiation Therapy

Zaremba et al. examined a total of 453 radiation cycles from Denmark in 2015. In all therapy-associated events (*n* = 14), the photon energy was ≥ 15 MV. The authors identified energy as the main risk factor, standing alone (odds ratio 5.73; 95% CI 1.58–20.76; [19]). Grant et al. found in a retrospective analysis of 249 treatment courses that RT with > 10 MV was the predominant risk factor for malfunction [14].

In a meta-analysis by Malawasi et al. in 2023, 32 studies with a total of 3121 patients were examined. In a cumulation of comparable studies, a significant association was found between event rate and higher energy > 10 MV, as well as increased susceptibility of ICDs. An event rate of 6.6% was described. A cumulative device dose of > 2 Gy had no relevant impact on the event rate [10]. Neutron-induced disturbances are primarily considered as the cause of these malfunctions. At high radiation energy (high linear energy transfer, LET), neutron production can lead to single-event upsets (SEU) in the circuits of the storage unit of an implanted device. These SEUs, as “soft errors,” do not physically damage the device, but they can lead to an electrical restart or loss of stored diagnostic data [20]. In the present study, there were no malfunctions or adverse events in patients treated with 6‑MV photons.

### Significance of device controls regarding malfunction

The alteration of aggregate parameters was examined by Bravo-James et al. in 2018, involving 109 patients. In two cases, a change in lead impedance (right atrium, right ventricle) occurred as a result of a “reset/restart.” These patients were treated with 6‑MV photons, with no consideration given to the dose to the device or lead insertion site. In two cases, a threshold increase in response to a restart was described (left ventricle, right ventricle). These events also involved radiation with 6 MV [21]. Overall, exceeding the predefined limits had no clinical relevance.

In the present study, even with a high cumulative dose of over 40 Gy at the lead insertion site and neutron-producing radiation, there was no significant change in lead impedance. The other relevant parameters such as threshold and perception were exceeded in 14.8% and 13.5%, respectively. In none of these cases was this change interpreted as problematic or result in a change in the course of therapy. While the limits of aggregate parameters have a fixed value in the consideration of the effects of radiological imaging (especially MRI imaging), a systematic consideration of these parameters in radiotherapy has not yet been firmly established in studies. The interpretation of a change in lead parameters must therefore be assessed by the attending cardiologist. A reliable proof of device malfunction cannot be derived. Notably, our data do not rule out late effects at the lead insertion site, as fibrosis usually takes months to years to develop.

Nevertheless, by collecting and analyzing aggregate parameters, in addition to secure malfunctions such as electrical restart and memory erasure, another objective control parameter is obtained. This may provide an opportunity to interpret an already collected and existing parameter and implement it into risk stratification. We were able to show that there is a considerable proportion of therapy courses with a temporary change in these parameters. Even though no correlation between this change and individual therapy plans was established in the small cohort, the question of the cause of this observation remains. In a prospective approach, these anomalies could be further investigated and also checked for differences between device types (pacemakers, ICDs). It should be noted that the change in parameters could not anticipate any adverse events or be directly related to them.

### Study limitations

The statistical power of the present study is limited by both the heterogeneous patient population and the small sample size. A generally cautious approach to radiation planning in these patients (avoidance of high radiation energies, low cumulative dose at the device) also before the guideline was published further leads to low event rates in the limited patient collective. Similar retrospective studies have also shown this limitation [10, 22].

However, the considered cohort is capable of further specifying national and international recommendations. In particular, the focus on reducing neutron-producing radiation to increase the safety of patients with implanted devices could be further supported.
